# The blame game- experiences of female residents in obstetrics & gynecology regarding lapses in patient safety

**DOI:** 10.12669/pjms.38.7.5741

**Published:** 2022

**Authors:** Shazia Tufail, Nilofar Mustafa, Rizwana Kamran, Junaid Sarfraz Khan

**Affiliations:** 1 Dr. Shazia Tufail, MCPS, FCPS, MHPE. Department of OB/GYN, CMH Lahore Medical College and Institute of Dentistry, (National University of Medical Sciences), Lahore, Pakistan; 2 Dr. Nilofar Mustafa, MCPS, FCPS, MHPE. Department of OB/GYN, CMH Lahore Medical College and Institute of Dentistry, (National University of Medical Sciences), Lahore, Pakistan; 3 Dr. Rizwana Kamran, MSc Medical education. School of Health Professions’ Education, CMH Lahore Medical College and Institute of Dentistry, (National University of Medical Sciences), Lahore, Pakistan; 4 Dr. Junaid Sarfraz Khan, PhD Medical Education. (UHS), PhD Medical Education Liverpool) School of Health Professions’ Education, CMH Lahore Medical College and Institute of Dentistry, (National University of Medical Sciences), Lahore, Pakistan

**Keywords:** Critical incidents, Medical errors, Patient safety, Patient harm, Surgical errors

## Abstract

**Objectives:**

Primary objective was to explore experiences of female residents of Obstetrics and Gynecology (OB/GYN) regarding lapses in patient safety (PS) while secondary objective was to explore factors hampering or favouring improvement of PS in OB/GYN.

**Methods:**

In this qualitative narrative study carried out in OB/GYN department of Combined Military Hospital (CMH) Lahore for six months from 1^st^ April to 30^th^ September 2021, six fourth-year residents were asked to write narratives of their personal experiences of medical error (witnessed or committed) in detail and reflect on those experiences, which were then transcribed. Code labels and themes were assigned manually. Interpretation of these themes was done after thematic content analysis.

**Results:**

Six, fourth-year female residents with a mean age of 28.6±1.8 participated in the study. Two main themes with sub-themes were identified: 1) Challenges in patient safety (Personal challenges, Workplace challenges, Barriers to PS), 2) Lessons learnt from experiences (Self-improvement and; Promotion of patient safety culture). Heavy workload with long working hours, lack of communication and teamwork, lack of experience and inadequate supervision were major factors involved in PS lapses experienced by residents.

**Conclusion:**

Incidents of Patient Safety (PS) lapses had a strong impact on the emotional and professional life of residents. Formal PS training with improvement of working conditions may help promote PS culture.

## INTRODUCTION

Safety culture is a complex phenomenon that consists of subcultures such as leadership, teamwork, evidence-based practices, communication, learning, and patient-centered practices.^1^ Data show that fifty percent of adverse events in healthcare are preventable.^2^ OB/GYN involves a dual high risk of both maternal and fetal morbidity and mortality, and requires a sound safety climate to prevent adverse outcomes. Medical skills and knowledge, and communication, both within the healthcare team and between healthcare professionals and patients, are vital contributors to patient safety. 2

Doctors, especially doctors-in-training such as residents, make errors.^3^,^4^ By definition, they are at a higher risk of committing errors during the stage of developing medical competencies.^5^,^6^ Residents deserve particular attention because behaviors learnt early in practice are likely to persist later in professional life.^3^,^4^ They have to face consequences even though today’s approach to errors emphasizes systemic factors.^5^

In his study, West noticed that self-perceived Patient Safety (PS) lapses were seen more when residents were stressed.^6^ In a study by Varjavand, it was seen that error disclosure and disclosure responsibility increased significantly over a decade when residents received formal training in PS.^7^ Causes for lapses in PS are multi-factorial. A comprehensive account of not only the documents and observers, but residents’ perceptions and perspectives should also be given due importance to gain full understanding of phenomenon.^8^ Furthermore, residents felt to be left alone in dealing with their first serious errors instead of a team management approach.^7^

This study was conducted with the aim to provide in-depth analysis of experiences of female residents in OB/GYN about medical errors and barriers to PS. Few data on resident doctors’ awareness and perception on PS come mainly from surveys.^3^,^4^ Considering these studies with their quantitative approach to a complex problem, current study was carried out to gather in-depth data from our own working environment to better understand experiences of female residents regarding medical errors. Primary objective of study was to explore experiences of female residents of OB/GYN regarding lapses in PS while secondary objective was to explore factors hampering or favouring improvement of PS in OB/GYN department. Research question of study was: What were the PS lapses experienced by OB/GYN residents and how could they have been avoided?

## METHODS

Study was started after approval from Ethical Review Committee (No 573/ERC/CMH/LMC dated 4-5-21). It was carried out for six months in the Department of OB/GYN of CMH Lahore from 1^st^ April to 30^th^ September, 2021. Fourth-year residents working in OB/GYN were included while male residents and residents not willing to participate were excluded from the study.

Fourth-year residents in OB/GYN department are team leaders of residents in CMH Lahore bearing responsibility of initial patient management. They are also responsible for liaison with attendants and staff. All six, fourth- year residents in OB/GYN department were voluntarily included through purposive sampling. Written informed consent was obtained from participants. Residents were counseled regarding purpose of study. It was informed that results of study will be published for educational purposes. They were requested to write a narrative of their personal experience of medical error (witnessed or committed) in detail and reflect on that experience. A set of questions was developed to help them write narrative in detail, based on literature review and discussion with three experts in OB/GYN and medical education. After acquiring the first narrative, set of questions was revised and another question added for clarification in the reflection segment. Residents were provided envelopes and requested to place their narratives in envelope. A staff member was designated to collect envelopes and hand over to the researchers in order to ensure anonymity.

**Fig.1:**
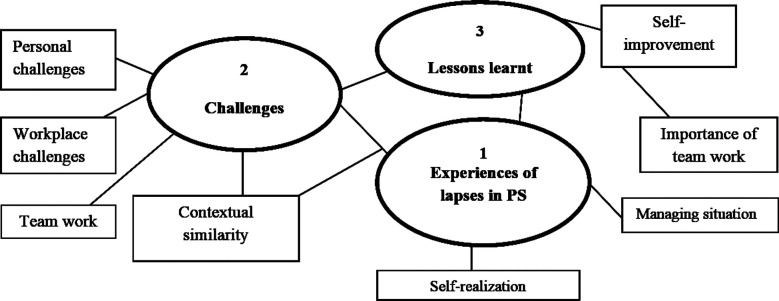
Initial thematic map developed with 3 main themes.

Narrative study design was chosen for study because it allows researcher to understand meaning-making of the residents’ experiences of lapses in PS.^9^ Thematic analysis was chosen as method of analysis as it provides an accessible and flexible interpretive approach “offering insight into meanings (themes) across a data set”.^10^ Narratives were transcribed and data analysis was done concurrently with data collection. Participants were given pseudonyms. Codes were assigned manually after repeated reading of the database, and then grouped together into broader themes serving as the key findings. An initial thematic map was developed based on relationship of various codes and themes (Fig.1). After further reading of collated extracts for each theme, two themes were finalized. In order to ensure credibility of study, methods of member checking and investigator triangulation were used. For member checking, summary of results was sent to all participants to see if it was based on participants’ narratives and not any potential bias of the researchers.^11^ Investigator triangulation was done by involving all co-researchers in every step of data collection and analysis.^11^Methodology was explained in detail in order to ensure dependability of study.^11^

## RESULTS

All six participants were fourth-year female residents working in OB/GYN department of CMH Lahore. They had been working in the hospital since the start of their training. Three of them were married. Their ages ranged between 27-33 years, mean age being 28.6±1.8 (Table-I).

**Table I:** Demographic characteristics of participants*.

S No	Name	Age (Years)	Marital status
1	Dr. Saira	27	Single
2	Dr. Hira	29	Married
3	Dr. Zareen	33	Married
4	Dr. Ifrah	26	Single
5	Dr. Tania	26	Single
6	Dr. Rania	31	Married

* Participants were given pseudonyms

### Two main themes were identified.

### Theme-1: Challenges in patient safety.

Three sub-themes were recognized (Table-II). Residents felt that there were multiple personal and workplace challenges they had to face during the critical incidents recalled. A contextual similarity was noted in their narratives. Almost all incidents experienced happened to involve high risk patients and situations arose at night time or early morning hours during the first year of residency. Residents felt that work schedules bearing long working hours (30 hours when doing on call duty) and exhaustive routine affected their ability in timely responding and coping with situations. Variable perceptions were found regarding the behavior of colleagues and seniors, with both positive and negative attitudes being witnessed by residents. Two residents voiced their concerns about the behavior of seniors who completely abandoned them and tried to put the blame on juniors. However, two residents appreciated the role and support of seniors. Residents experienced mental trauma and self-guilt following critical incidents. They felt that in addition to inexperience, lack of understanding the gravity of situation and poor communication with peers and seniors, logistic issues at the workplace and lack of continuity of care were also contributory. One resident attributed lapses to “lack of evidence-based practices and standardized protocols”. Interestingly, majority of residents were found to be unaware of importance of formal PS training, its lack at undergraduate and postgraduate level being at core of such lapses.

**Table II:** Final themes with sub-themes, codes and selected quotes of participants

	Themes	Sub-themes	Codes	Representative Quotes
I	Challenges in PS	Personal challenges	Self-guilt	“I was at end of 1^st^ year training, and was on night call. A primigravida was in 2^nd^ stage in labour room at about 0300 hrs in the morning for past 2 hrs.” (Zareen) “I felt guilty, terrible and cried for days.” (Hira)
			Time Management	
			Lack of experience	
		Workplace challenges	Role of seniors/colleagues	“An inquiry was held,…and there I saw the blame game. First on call was lying to put the whole burden of situation on junior residents. I was told you should have called the first on call yourself.” (Rania) “My seniors scolded me for missing the CTG trace at 3am, making me feel like the whole mishap was my doing.” (Hira) “We trust more on the pass it down practices then evidence based practices.” (Ifrah) “I always knew that counselling is a huge part of our job but that day, I was impressed with our consultants.” (Saira)
			Working hours	
			Lack of evidence based practices	
			Issues in logistics	
			High risk patients	
		Barriers to PS	Irresponsible attitude	“I tried to wake up my senior to make a decision for episiotomy or cesarean section but due to round the clock hectic routine since morning, she couldn’t wake up in time. Somehow, I did episiotomy and managed to deliver the baby, but mother developed 3^rd^ degree perineal tears.” (Zareen)
			Communication gaps	
			Lack of PS training	
II	Lessons learnt from experiences	Self-improvement	Overcoming guilt and trauma	“It was one of the most mentally traumatic experience in the very start of my training life. I decided to work onwards in an organized manner, doing justice to myself and my profession with specified timings in hospital.” (Tania) “After that, I am very vigilant about patient safety before, during and after any surgical procedure.” (Ifrah)
			Self-directed learning about PS	
			Improving professional competencies	
		Promotion of PS culture	Role of team work	“We need to work as a team, trusting each other and dividing our work, following evidence based practices and helping each other.” (Hira)
			Standardized protocols needed	
			Well-being of residents	

### Theme-2: Lessons learnt from experience.

Two sub-themes were recognized (Table-II). Residents took incidents as an inciting factor to improve their competencies. Although initially they faced difficulty in overcoming mental trauma and self-guilt following critical incidents, they later used these as strength to improve themselves. They thought that team work was essential to avoid such lapses; colleagues and seniors being perceived as those who could help them in overcoming their deficiencies and improving their communication and procedural skills. Only one resident felt that PS culture should be created with standardized protocols, regular drills and training in various PS issues. Residents expressed their concerns about working conditions with improvement being needed in working hours and schedules. Residents thought that importance should be given to overall well-being of residents to keep them mentally at peace and ready to cope with any sort of emergency situation.

## DISCUSSION

The study gained important insights into factors associated with lapses in PS. There was contextual similarity in incidents which typically occurred at night or early morning hours when residents were exhausted after almost 24 hours long duty (Table-II). Findings are comparable to a study carried out by Kalmbach in which comparatively higher rate of lapses in PS was found in residents sleeping <6 hours per night.^12^ Reduced sleep and long working hours affect overall health and well-being of residents, ultimately affecting their performance.^12^ The study conducted by Coffyn and Seingsukon in 2020 also corroborated this when they studied Doctor of Physical Therapy students.^13^

Half of the residents were married with additional responsibilities of caring for their families after working hours. However, they felt that they were not being well looked-after and were expected to be some super-humans, as Dr. Hira explained, “Once you become a doctor you are no more human who needs sleep, food and self-care”.

Residents were of opinion that they tried to support each other in this challenging field and hectic schedule. Their working conditions should be such that they can perform their best in a favorable environment and are able to cope with emergencies timely in a professional manner.^14^,^15^ In contrast, Beckman in 2012 found that well-being of residents is not related to clinical performance of residents.^16^ However, his study involved assessment of knowledge and clinical performance and not the real setting incidents.

Other important barriers to PS identified by study included hesitancy and fear of seeking advice from seniors. Instead, they relied on their immediate senior resident for advice and support which was not always available (Table-II). This attitude depicts communication gap and ineffective teamwork which resulted in medical errors in two incidents reported by residents. These findings are in line with a local study in which inexperience and not seeking advice were found to be among the significant factors affecting PS climate.^17^ Residents also took responsibility of managing high risk patients (which was out of their domain) without informing seniors and this irresponsible attitude later on resulted in harm to patients. Handling of complex cases was also one of the common causes of surgical errors in a local study.^17^ Also, during the first year, the residents were sometimes not fully aware of logistics of hospital which resulted in undue delays in emergency patient management.^14^

Residents expressed that colleagues and seniors should be easily approachable and fully assess situation in case of any untoward incident before blaming the junior most residents. Tendency to blame healthcare professionals unnecessarily was also seen in a meta-synthesis of qualitative studies on PS done in 2015.^18^ Such behavior results in lowering morale of residents. In all incidents, residents faced severe mental trauma and feelings of self-guilt which took a long time to heal (Table-II). Findings are comparable to those of a qualitative study carried out on internal medicine female residents.^5^ Similar findings are seen in contemporary studies in which the residents go through a complex range of emotions after an adverse incident.^17^,^19^ However, after recovering from initial mental trauma, eventually they were all able to use those incidences as life changing experiences and improved themselves through self-directed learning. They enhanced their time management, communication skills and procedural skills. All of them expressed that incidents had a major positive impact on their lives helping them to grow professionally. This is in contrast to the findings of White and Gallagher who found that the residents behave differently after an adverse event, and may even lose their self-confidence.^19^

**Table:** APPENDIX A--- QUESTIONS

**A-Personal experience regarding lapse in patient safety:**
1. Could you describe this experience and explain how you were involved?
2. If you think again about that time, do you remember you felt?
3. How did your supervisors, colleagues and relatives react at that time?
4. Did you feel supported by your supervisors and colleagues?
5. Which were the main factors that contributed to this error?
**B-Reflection:**
1. Today, how do you feel about this error?
2. How did this error impact on your private and professional life?
3. Were there long-term consequences?
4. Which resources did you use to manage the error?
5. Could you have used other resources?

Residents felt that lapses in PS can be reduced through effective team work (Table-II). Findings are comparable to those of a study done to explore factors associated with PS climate in which team work was identified as one of the major factors associated with improving PS culture.^20^ Another recent study carried out in Brazil also corroborated findings where teamwork was found to have a strong positive influence on PS.^21^

Overall, the study reflected lack of formal PS training of the residents to be a major cause of these incidents. It was surprising that none of them realized the need for PS training despite two participants mentioning need for standardized protocols. Results are in contrast to a local study regarding perceptions of medical students about PS where students were found to be highly in favour of PS teaching and training.^22^ A recent study carried out in Istanbul also stressed the importance of PS training in creation of PS culture.^23^ Efforts to improve PS climate through innovative training and standardized protocols in an obstetric unit also resulted in significant improvement over five years from 2004-2009.^24^

### Limitations

The study was exploratory, qualitative research which is limited by small number of participants of a single gender to keep the data at a manageable level. It depended on information given by participants which could have been affected by their honesty and willingness and ability to recall and fully articulate their experiences. Results were not meant to be generalizable; rather they provide an in-depth exploration of perspective of female residents of a particular military hospital. To our knowledge, current study is the first qualitative study done to explore the phenomenon in our local context. Further larger scale, multiple source studies with comparison of both male and female residents working in different contexts (military, civil, and private hospitals) are needed in order to endorse the findings of present study.

## CONCLUSION

Nurturing a PS culture helps not only in improving patient care but also benefits the institution by reducing expense due to medical errors and helps build trust with community.^25^ Results of this study could be used in formulating plans and Standard Operating Procedures (SOPs) to improve the training of postgraduate residents in responding and coping with medical errors. Improvement in PS will help in better patient care in department and the organization overall.

### Authors’ Contribution:

**ST & JSK** Conceived and designed the study and performed analysis and editing of manuscript and both are responsible and accountable for the accuracy and integrity of research.

**ST, NM & RK:** Did data collection and manuscript writing and all authors did review and final approval of manuscript.
